# Perioperative Glycaemic Control and Orthopaedic Outcomes: A Narrative Review of Evidence and Future Directions

**DOI:** 10.7759/cureus.96905

**Published:** 2025-11-15

**Authors:** Samson Arokiyanathan, Harine Baalamurugan, Ignatius Ip, Leora Marcus, David Mafullul, Janthula Ranchagoda

**Affiliations:** 1 Orthopaedics, West Hertfordshire Teaching Hospitals NHS Trust, Watford, GBR; 2 Medicine, West Hertfordshire Teaching Hospitals NHS Trust, Watford, GBR; 3 Anaesthesiology, West Hertfordshire Teaching Hospitals NHS Trust, Watford, GBR

**Keywords:** diabete mellitus, glycaemic targets, orthopaedics surgery, perioperative glucose, total joint arthroplasty

## Abstract

In orthopaedic surgery, diabetes mellitus (DM) is consistently associated with delayed fracture healing, non-union, implant failure, periprosthetic joint infection, and poorer postoperative recovery.

Although strict perioperative glycaemic control has been shown to improve outcomes in general surgical populations, current national thresholds are largely extrapolated from non-orthopaedic data and focus primarily on cardiovascular and soft-tissue complications rather than bone biology. Existing recommendations from national guidelines vary and remain non-specific to orthopaedics. No consensus exists on optimal perioperative glucose or glycated haemoglobin (HbA1c) targets for elective orthopaedic interventions.

This review evaluates current evidence linking perioperative glycaemic status to orthopaedic outcomes. Relevant literature, particularly focused on total joint arthroplasty (TJA), was identified through a comprehensive search of PubMed, MEDLINE, and Google Scholar. Evidence reviewed in this article suggests that perioperative hyperglycaemia, rather than HbA1c alone, is a stronger predictor of complications.

Prospective studies are needed to assess preoperative HbA1c and perioperative glucose trajectories and their relationship with standardised outcomes. The potential harms of intensive control, such as hypoglycaemia and surgical delays, also require careful consideration. Additionally, randomised controlled trials (RCTs) comparing intensified versus standard glucose control protocols are required, with both efficacy and safety as endpoints. Defining evidence-based, orthopaedic-specific glycaemic thresholds is essential to reduce complications, enhance recovery, and improve long-term outcomes.

## Introduction and background

Diabetes mellitus (DM) is a chronic metabolic condition with an ever-growing prevalence in today’s modern world. Complications of diabetes extend far beyond its metabolic manifestations and have consequences not only on impaired bone healing but also on surgical site infections, implant failure, and delayed fracture union/non-union. Studies have shown that hyperglycaemia in individuals with DM contributes to these poor outcomes through microvascular dysfunction, oxidative stress, and the accumulation of advanced glycation end-products (AGEs) [1]. Optimisation of peri-operative glycaemic targets has been associated with improved postoperative outcomes; however, national guidelines are not based on orthopaedic studies, and therefore specific thresholds for glycaemic control in orthopaedic interventions remain inadequately defined. This review critically examines the impact of suboptimal glycaemic control on postoperative outcomes in orthopaedic surgery, with a particular focus on lower-limb total joint arthroplasty where the majority of available evidence exists, and proposes evidence-based perioperative glycaemic targets to inform clinical practice.

Objective

This review aims to explore the current evidence linking glycaemic control with postoperative orthopaedic complications and to evaluate existing literature. It also aims to interrogate current national guidance while outlining research priorities for establishing optimal perioperative glycaemic control, and to propose perioperative glycaemic targets for future elective orthopaedic procedures.

Pathophysiology of diabetes on bone

DM is a metabolic disorder that adversely affects bone health, leading to an increased risk of fractures, impaired bone formation, and delayed fracture healing. Chronic hyperglycaemia and the resulting accumulation of AGEs, elevated reactive oxygen species (ROS), and persistent inflammation disrupt normal bone remodelling processes. These metabolic and inflammatory disturbances alter the balance between bone-forming osteoblasts and bone-resorbing osteoclasts, promoting osteoclast activity while reducing osteoblast number and function [2].

Several studies have demonstrated associations between AGE accumulation in bone tissue and disruption of both cellular function and microarchitecture. Formed through non-enzymatic reactions between glucose and structural proteins or lipids under chronic hyperglycaemia, AGEs induce excessive collagen cross-linking, leading to reduced bone elasticity and increased fragility [3]. Beyond these matrix effects, AGE binding to the receptor for advanced glycation end-products (RAGE) triggers oxidative stress and inflammatory signalling, promoting osteoclastogenesis while inhibiting osteoblast activity [4]. At the bone-implant interface, these processes impair osseointegration through collagen degradation and persistent inflammation, thereby increasing the risk of aseptic loosening and implant failure [3].

In DM, pro-inflammatory mediators such as TNF-alpha, IL-1β, IL-6, and IL-18 are elevated locally and contribute to diabetic complications. TNF-alpha, in particular, is a major cytokine contributing to insulin resistance [2]. It alters lipid metabolism, increasing free fatty acids that promote skeletal muscle insulin resistance and hepatic gluconeogenesis. It also disrupts insulin signalling by suppressing insulin receptor tyrosine kinase activity and reducing Insulin Receptor Substrate-1 (IRS-1) phosphorylation, leading to decreased cellular insulin sensitivity [5]. Diabetic individuals exhibit an impaired ability to downregulate inflammation once triggered. Elevated TNF levels may also hinder suppression of other inflammatory genes and promote apoptosis, resulting in disrupted bone remodelling and impaired coupling.

Diabetes increases ROS production through mitochondrial superoxide generation, nicotinamide adenine dinucleotide phosphate (NADPH) oxidase activity, and stimulation of AGE-related pathways, while simultaneously reducing antioxidant defences [2]. This oxidative stress promotes RANKL expression and osteoclast differentiation, resulting in bone resorption. FOXO transcription factors protect against oxidative damage by inducing antioxidant enzymes, but dysregulation of this process in DM enhances oxidative stress and osteoclastogenesis [6]. Overall, these processes alter bone biology and have been shown to disrupt osseointegration by way of osteoblast impairment, enhanced osteoclast activity, and compromised bone quality, thereby jeopardising implant integration in DM patients.

Study selection

Our narrative review was conducted using the National Library of Medicine databases (PubMed/MEDLINE/Google Scholar) to identify English-language articles published from January 1, 2005, to May 5, 2025.

The search strategy used the following terms: (“diabetes mellitus” OR “hyperglycaemia” OR “HbA1c”) AND (“arthroplasty”)

Filters were applied to include human studies, adult populations (≥18 years), and English-language publications. Reference lists of relevant articles were also manually screened to identify additional eligible studies.

Exclusion criteria comprised case reports, small case series (<50 patients), studies lacking orthopaedic-specific outcomes, and non-English publications. After duplicate removal, 268 titles and abstracts were screened, of which six key articles met the inclusion criteria. Screening of reference lists yielded one additional study. Included articles were selected based on their relevance to clinical outcomes, perioperative glycaemic management, and mechanistic insights into bone health and implant integration.

Data extraction focused on study design, cohort comparability, outcome assessment, and adequacy of follow-up. While no formal quantitative scoring system was applied, studies with a high risk of selection or reporting bias were interpreted with appropriate caution.

Evidence was synthesised qualitatively, highlighting key trends, areas of controversy, and gaps in the existing literature.

## Review

Comparison of current guidelines 

The current glycaemic targets in the UK (NICE), Europe, and the US (American Diabetes Association (ADA)) are non-specific and largely informed by data from general surgical and medical populations [7-11]. These guidelines were introduced primarily to prevent cardiovascular complications, impaired wound healing, and systemic infection. However, orthopaedic surgery presents qualitatively different challenges that have not been considered when establishing the above guidance (Table 1). Perioperative glycaemic control targets recommended by both NICE and Centre for Perioperative Care (CPOC) are based on approximately 30 randomised controlled trials (RCTs), most of which are cardiothoracic or general-surgery focused [9-10]. There are almost no orthopaedic studies involved in setting these targets. The committee explicitly notes that the 6-10 mmol/L range was not the direct focus of trials [9], and there is no orthopaedic-specific RCT in the NG180 review that defines a unique glucose target [9]. Furthermore, these recommendations lack clarity on the perioperative period itself, instead focusing on long-term diabetes management. The vagueness of perioperative targets, for example, “optimise glycaemic targets where possible” [8], results in substantial variation in clinical practice. The lack of alignment between guideline evidence bases and orthopaedic outcomes underscores the need for dedicated research to establish specialty-specific glycaemic recommendations.

**Table 1:** Current guidance and perioperative targets. Presentation of perioperative CBG targets recommended by leading international organisations for adults with diabetes undergoing surgery. The ADA and the Association of Anaesthetists [7-8] propose explicit glycaemic ranges, whereas UK-based bodies such as NICE, the Joint British Diabetes Society/CPOC, and Diabetes UK [9-11] place greater emphasis on monitoring and holistic care rather than fixed CBG or HbA1c thresholds. ADA: American Diabetes Association; CBG: Capillary Blood Glucose; NICE: National Institute for Health and Care Excellence; JBDS: Joint British Diabetes Society; CPOC: Centre for Perioperative Care.

Guideline / Source	Recommended Perioperative CBG Targets
ADA (USA)	80-180 mg/dL (4.4-10.0 mmol/L)
Association of Anaesthetists	6-10 mmol/L
NICE (UK)	Routine testing; no strict threshold
JBDS / CPOC (UK)	Focus on daily glucose (6-10 mmol/L)
CPOC / Diabetes UK	Holistic care guidance; no specific CBG or HbA1c target for bone healing or union outcomes

In orthopaedic surgery, the consequences of hyperglycaemia extend directly to bone repair and osseointegration, processes unique to this specialty, and clinical outcomes such as non-union, delayed union, implant loosening, and periprosthetic joint infections (PJI) may have long-term functional consequences. NICE guidance demonstrates that patients with DM undergoing abdominal surgery have an elevated risk of wound infection and cardiopulmonary complications but no consistent effect on tissue-specific healing beyond soft-tissue repair [9]. In contrast, in orthopaedic patients, the risk of biological healing failure is substantial, with diabetes associated with up to a twofold increased risk of fracture non-union [12]. These risks are not only higher but also mechanistically distinct, reflecting the effects of impaired angiogenesis, osteoblast dysfunction, and altered collagen maturation in DM patients [13].The differences in outcomes such as these suggest that applying generic preoperative HbA1c targets to orthopaedic patients may be insufficient. While such targets may still help mitigate systemic complications, they do not address the specific risk of impaired bone healing and therefore may not be fully generalisable to orthopaedic practice.

Association between diabetes and adverse outcomes in orthopaedic populations

Multiple large-scale studies have established diabetes mellitus (DM) as an independent predictor of adverse postoperative outcomes in orthopaedic surgery. Reported associations include delayed or failed fracture union, increased susceptibility to infection and PJI, inferior functional recovery, and elevated rates of revision surgery.

A systematic review and meta-analysis published in 2020 found that individuals with diabetes had approximately 2.11-fold higher odds (95% CI: 1.33-3.37; p = 0.002) of experiencing delayed union, non-union, or malunion compared with non-diabetic individuals [12]. This study played an important role in attempting to quantify the impact of diabetes using the currently available evidence. The need for strict glycaemic control in perioperative patients, given its direct effect on fracture healing, is emphasised throughout the study. However, it is limited by the fact that all data are derived from retrospective studies, highlighting the lack of RCTs. Further to this, several disparities exist across the studies included in the review, such as the level of blood glucose control, diabetic treatment regimens, and inconsistencies in definitions of non-union, all of which introduce significant unaccounted heterogeneity. Despite these limitations, the study’s conclusion regarding the increased risk of impaired fracture healing warrants further research.

A retrospective study by Reich MS et al. investigated the impact of diabetic control on the risk of surgical site infections (SSIs) in orthopaedic trauma patients [14]. The authors found that poorly controlled diabetes, characterised by elevated HbA1c or poor perioperative glycaemic control, significantly increased the risk of SSIs post-surgery. While this study provides valuable insights into the importance of glycaemic management in preventing infection, similar to the limitations seen in the studies reviewed by Ding ZC et al. [12], its retrospective design introduces potential biases such as selection and information bias. Additionally, the study may be limited by unaccounted confounders such as smoking or nutritional status, and by the inability to establish causality, as it only demonstrates an association between diabetic control and infection risk. Despite these limitations, the findings further reaffirm the importance of preoperative glycaemic control. Prospective studies with more rigorous controls and longer follow-up are needed to confirm these results.

Substantially higher infection risk in postoperative orthopaedic patients, particularly following arthroplasty, has also been demonstrated. A systematic review of primary total knee arthroplasty (TKA) encompassing over 119,000 patients revealed that diabetic patients experienced a 1.84-fold higher risk of infection compared with non-diabetic patients [15]. Within this review, Ahmad MA et al. observed a slightly lower prevalence of superficial surgical site infections (SSSIs) among patients with diabetes compared with non-diabetic individuals; however, this difference did not reach statistical significance. Interpretation of this finding is constrained by the limited evidence base, as only two studies within the review specifically reported outcomes related to SSSIs, thereby restricting the reliability of this comparison. Iorio R et al. conducted a retrospective analysis of 4,241 total joint arthroplasties to evaluate postoperative infection patterns in diabetic and non-diabetic patients. The incidence of infection was significantly higher among individuals with diabetes; however, most cases were deep infections involving the fascia or joint capsule. Only 18 superficial infections were reported in total (nine cellulitic and nine operative abscesses), indicating that deep periprosthetic infections account for the predominant infectious burden in this cohort [16]. Overall, the evidence supports an increased susceptibility to postoperative infections among diabetic patients undergoing arthroplasty, although the influence on superficial infections remains less well-defined.

A meta-analysis including 16 studies on PJI following hip or knee arthroplasty reported an adjusted OR of 1.58 for patients with DM (95% CI: 1.37-1.81) [17]. This risk has been further demonstrated in another meta-analysis of patients who underwent TKA, which reported that DM was associated with a RR of 1.71 (95% CI: 1.46-2.00) for PJI, with follow-up-stratified RRs of 1.40 (<1 year) and 2.23 (>1 year) [18]. This study also demonstrated that prosthetic revision rates were higher in diabetic patients (RR 1.37; 95% CI: 1.23-1.52). Furthermore, patients who were hyperglycaemic in the peri-operative period, even in the absence of a formal DM diagnosis, were found to have a higher incidence of SSI and osteomyelitis.

These studies emphasise the impact that poor glycaemic control can have on early orthopaedic outcomes, not just long-term fracture healing. In addition to biological outcomes, patients with DM have been shown to have poorer functional outcomes after orthopaedic procedures. Studies have demonstrated higher rates of readmission, reoperation, and revision surgery in diabetic cohorts compared with non-diabetic patients [19]. Recovery trajectories are also slower, with DM patients reporting lower postoperative mobility scores and delayed return to baseline function, both of which can significantly impact quality of life.

Review of glycaemic targets in lower limb total joint arthroplasty

It is well established that a significant proportion of patients undergoing total joint arthroplasty have poorly controlled glycaemia, with recent studies highlighting a strong association between poor glycaemic regulation and increased postoperative complications in TJA specifically. Patients with DM undergoing joint replacement surgeries are consistently shown to be at higher risk for complications such as osseointegration failure and SSIs. The underlying pathophysiology is believed to stem from impaired osteoblast function, reduced collagen synthesis, and disruptions in bone remodelling, as outlined earlier. These impairments in bone healing and integration often result in failed implant-bone bonding, leading to aseptic loosening and joint instability, ultimately necessitating revision surgeries. As highlighted previously, substantial evidence demonstrates poor postoperative outcomes in DM patients; however, strict glycaemic targets, whether HbA1c-based or peri-operative CBG targets, have yet to be clearly established.

A retrospective study of 6,088 diabetic patients undergoing total hip or knee arthroplasty found that those with HbA1c ≥ 7% had a significantly higher RR of postoperative complications compared with those with better glycaemic control [20]. However, with respect to complications affecting the prosthetic joint, no significant association between elevated HbA1c and PJI was found. Harris AH et al. also acknowledged important limitations, noting that the retrospective design introduced heterogeneity, the cohort was disproportionately male, and potential confounding variables were not adequately controlled.

Godshaw BM et al. conducted a retrospective analysis of 773 diabetic patients undergoing total hip or knee arthroplasty at a single institution to evaluate the relationship between preoperative HbA1c levels, perioperative glycaemic control, and risk of prosthetic joint infection [21]. The study demonstrated that patients with HbA1c exceeding 7.45% were significantly more likely to experience postoperative hyperglycaemia (>200 mg/dL), although no statistically significant association with infection risk was observed. In addition, Iorio et al. reported that preoperative HbA1c levels were not a reliable indicator of postoperative infection risk in total joint arthroplasty, with no statistically significant association observed between elevated HbA1c and infection incidence (p = 0.293) [16]. This finding suggests that HbA1c alone is insufficient as a predictive marker of postoperative infectious complications. The retrospective design further limits causal interpretation, as potential confounding variables may have influenced the outcomes observed.

Chrastil J et al. investigated the link between HbA1c and PJI in TJA by conducting a large retrospective study of 13,272 patients [22]. Similar to previous studies [20-21], Chrastil J et al. did not find a significant association between HbA1c and risk of PJI; however, this study did detect a strong predictive association between peri-operative hyperglycaemia and PJI. The study also reported a 30% increase in two-year postoperative mortality among patients with poorly controlled diabetes, although a causal association has not been established, limiting the strength of this finding.

Kremers HM et al. (2015) conducted a large retrospective cohort study involving 20,171 primary and revision total joint arthroplasties using data from the Rochester Epidemiology Project to examine the relationships between diabetes, perioperative hyperglycaemia, and postoperative complications [23]. The study incorporated longitudinal follow-up to evaluate the incidence of prosthetic joint infection (PJI), aseptic loosening, and revision surgery. Consistent with the findings of Chrastil J et al. [22], no significant association was observed between HbA1c (whether analysed as a continuous variable or using a 7% threshold) and the risk of PJI. Both preoperative and postoperative hyperglycaemia were significantly associated with an increased incidence of infection. Notably, elevated perioperative glucose levels were also linked to a higher, albeit non-significant, risk of PJI among non-diabetic patients, suggesting that acute hyperglycaemia may influence infection risk independently of diabetic status.

Kremers HM et al. (2017) further expanded on their earlier study by examining associations between preoperative hyperglycaemia and the risk of aseptic loosening in TJA patients [24]. In the multivariate-adjusted analysis, elevated preoperative hyperglycaemia (cut-off >180 mg/dL) was independently associated with an increased overall risk of revision and revision secondary to aseptic loosening among diabetic patients. These findings imply the importance of glycaemic control and demonstrate how perioperative hyperglycaemia may adversely affect osseointegration and bone remodelling, predisposing implants to failure regardless of infection status.

Postoperative hyperglycaemia in elective general surgical patients has been shown to be strongly predictive of nosocomial infections [25]. Most recommendations made by NICE and CPOC are indeed based on evidence from non-orthopaedic populations. Kheir MM et al. examined the association between postoperative hyperglycaemia and prosthetic joint infection, identifying a linear relationship between glucose levels and infection risk, and proposing a glycaemic threshold of 137 mg/dL to minimise complications [26]. However, similar to previous retrospective single-institution studies, it was limited by potential biases and lacked standardisation of glucose measurements (e.g., fasting vs. non-fasting morning values).

Overall, the evidence indicates that perioperative hyperglycaemia may be a more immediate and clinically meaningful predictor of poor orthopaedic outcomes than HbA1c alone. However, most studies to date are retrospective and single-centre, with inherent methodological limitations, underscoring the need for prospective, orthopaedic-specific research to define clear, evidence-based glycaemic targets. Based on the above findings, there is an inadequate link between HbA1c thresholds and adverse outcomes; therefore, a specific HbA1c target cannot be proposed. This is particularly significant given that current perioperative national guidelines often rely on HbA1c thresholds, suggesting a need for a shift in national guidance to incorporate perioperative CBG targets instead. A perioperative CBG threshold of 110-150 mg/dL can be suggested to minimise infection risk while avoiding hypoglycaemia (Table 2).

**Table 2:** Summary of studies examining the relationship between glycaemic control and surgical outcomes in TJA. TKA: Total Knee Arthroplasty; THA: Total Hip Arthroplasty; TJA: Total Joint Arthroplasty; DM: Diabetes Mellitus; PJI: Periprosthetic Joint Infection; SSI: Surgical Site Infection; HbA1c: Glycated Haemoglobin; mg/dL: Milligrams per Decilitre.

Author	Year	Study design	Sample	Procedure	Key results	Comment
Harris AH et al. [20]	2013	Retrospective cohort	6,088	TKA/THA	Presurgical HbA1c ≥7% was associated with an elevated risk of having at least one surgical complication. The increase in risk from 7.1% to 8.7% is small in absolute terms but larger in relative terms. No significant association between HbA1c and PJI.	Sample consisted mostly of male participants. Patients with poorer diabetic control likely had more recorded and more recent HbA1c values; therefore, more patients with better control may have been excluded.
Godshaw BM et al. [21]	2018	Retrospective chart review	773	TKA/THA	HbA1c >7.45% resulted in greater likelihood of postoperative glucose >200 mg/dL. PJI did not statistically increase with HbA1c.	Retrospective design limits data. Study significantly underpowered to assess PJI. No non-diabetic control cohort.
Kheir MM et al. [26]	2018	Retrospective review	24,857 (13,198 with ≥1-year follow-up)	TKA/THA	Rate of PJI increased proportionally from blood glucose ≥115 mg/dL. Significant association between glucose levels and PJI (p = 0.028). Optimal blood glucose threshold to reduce PJI risk was 137 mg/dL.	Retrospective design; glucose levels not consistently fasting. Protocol changes occurred over the 15-year study period.
Chrastil J et al. [22]	2015	Retrospective cohort	13,272	THA/TKA	Preoperative glucose levels correlated with a significantly increased risk of PJI with an optimal cut-off ≥194 mg/dL. No significant correlation between HbA1c and PJI.	Mostly male sample. Patients with poorer diabetic control more likely to have HbA1c recorded; patients with better control may have been underrepresented.
Kremers HM et al. [23]	2015	Retrospective cohort	20,171	THA/TKA	Significant association between PJI and perioperative hyperglycaemia (>180 mg/dL) when adjusted for age and gender. Associations became non-significant after accounting for confounders. No significant association between HbA1c and PJI.	Retrospective design with multiple confounders.
Iorio R et al. [16]	2012	Retrospective cohort	3,468	Primary or revision THA/TKA	HbA1c was not associated with superficial or deep surgical site infection. Diabetic patients had a significantly higher overall risk of infection after TJA.	Infection incidence was higher in DM patients, with deep periprosthetic infections accounting for the predominant burden.
Kremers HM et al. [24]	2017	Retrospective cohort	2,911	THA/TKA	Higher preoperative glucose values on the day before surgery were significantly associated with overall revision risk and revision for aseptic loosening. No significant association between HbA1c and PJI.	Confounded by variables such as age, gender, BMI, and procedure type.

Limitations and future research priorities

In this review, we have established that the current evidence base linking perioperative glycaemic control with fracture healing is limited and heterogeneous. Most available studies are retrospective cohorts, often with small sample sizes, variable follow-up periods, and multiple confounding factors (e.g., timing of HbA1c measurement prior to surgery, duration of surgery, differing definitions of hyperglycaemia and PJIs/SSIs). This lack of standardisation makes comparisons across studies challenging. Furthermore, while numerous investigations have demonstrated an association between diabetes or perioperative hyperglycaemia and adverse postoperative outcomes, very few have directly correlated preoperative glycaemic indices, such as HbA1c, admission glucose, or perioperative glucose variability, with radiographic evidence of osseointegration.

The potential risks of aggressive glucose management are underexplored. While tighter perioperative glycaemic control may reduce infection risk and improve healing, it inevitably increases the risk of hypoglycaemia, which is particularly dangerous in the surgical setting, where symptoms may be masked by anaesthesia or analgesia. In a cardiac surgery cohort, Johnston LE et al. identified postoperative hypoglycaemia as a predictor of increased operative mortality and morbidity, underscoring the potential risks of overly intensive glycaemic control [27]. Adherence to strict glycaemic thresholds, particularly long-term markers such as HbA1c, may also unnecessarily delay surgery and thereby worsen patient outcomes, negating potential benefits of euglycaemia. A retrospective study by Giori NJ et al. reviewed 404 diabetic patients scheduled for primary total joint arthroplasty to assess whether surgery was delayed due to a high HbA1c, and if so, for how long. They found that the average time required to achieve an HbA1c <7% was 8 months, and in some cases up to 3 years [28]. Harris AH et al. further demonstrated that applying an HbA1c threshold of 7% as a criterion for surgical eligibility would have resulted in unnecessary delays for approximately 31.9% of patients, most of whom would not have experienced postoperative complications had their surgery proceeded as planned [20]. Such deferrals not only impose additional physical and psychological strain on patients but also contribute to avoidable socioeconomic and healthcare system burdens. It is also important to note that routine HbA1c testing has the potential to identify previously undiagnosed diabetes. Current NICE and CPOC perioperative guidelines recommend, although do not mandate, HbA1c testing in patients with known or suspected diabetes prior to surgery [9-10]. Such mandates could potentially facilitate both the detection of unrecognised cases and the optimisation and follow-up of those with established disease.

While this review has focused primarily on elective arthroplasty patients, it is important to consider trauma patients, who may also benefit from perioperative glycaemic optimisation. Richards JE et al. demonstrated, using multivariable logistic regression, that having two or more blood glucose measurements exceeding 200 mg/dL was a significant risk factor for SSIs within 30 days postoperatively in patients with traumatic open fractures [29]. This highlights the potential value of defining trauma-specific glycaemic targets, particularly for identifying high-risk individuals and optimising interventions such as prophylactic antibiotic regimens or antibiotic-impregnated implants. This review has concentrated on the consequences of hyperglycaemia in TJA patients, as prospective studies in this population are more feasible due to the ability to plan interventions in advance. In contrast, trauma patients face a higher risk of hypoglycaemia and other adverse events when strict glycaemic control is attempted acutely, which may compromise outcomes. Additionally, this review does not specifically address upper-limb arthroplasty due to a relative paucity of studies evaluating the effects of glycaemic control on outcomes, underscoring a clear need for further research.

These limitations and previously highlighted literature gaps denote the need for dedicated orthopaedic research. Large prospective cohort studies should systematically collect preoperative HbA1c values and perioperative glucose trajectories, including time-in-range metrics from continuous monitoring, and correlate these with standardised definitions of fracture union and non-union, infection, revision, and functional outcomes. Randomised controlled trials are required to determine whether intensified perioperative glucose protocols improve bone healing compared with standard care, with hypoglycaemic risk as a key secondary outcome. Health-economic and implementation studies should evaluate the cost-effectiveness and practical challenges of preoperative optimisation. Only with such targeted research can evidence-based, orthopaedic-specific glycaemic thresholds be established to improve patient safety and postoperative outcomes.

## Conclusions

Diabetes mellitus is strongly associated with poorer outcomes after orthopaedic procedures. Yet, current perioperative glycaemic targets are based largely on general surgical populations, focusing on cardiovascular and soft-tissue complications rather than bone biology. The use of HbA1c thresholds in TJA has shown inconsistent associations with PJIs and postoperative outcomes, whereas perioperative hyperglycaemia assessed by CBG measurements has demonstrated a more consistent correlation with adverse events. Current national guidance remains centred on HbA1c levels and does not consider perioperative hyperglycaemia as a necessary parameter for optimisation. Dedicated prospective studies and randomised controlled trials are therefore needed to define safe, orthopaedic-specific perioperative glycaemic targets that balance the benefits of improved bone healing against the risks of intensive management, ultimately improving long-term outcomes for this vulnerable patient group.

## References

[1] Brownlee M (2005). The pathobiology of diabetic complications: a unifying mechanism. Diabetes.

[2] Jiao H, Xiao E, Graves DT (2015). Diabetes and Its Effect on Bone and Fracture Healing. Curr Osteoporos Rep.

[3] Henderson S, Ibe I, Cahill S, Chung Y-H, Lee F (2019). Bone quality and fracture-healing in type-1 and type-2 diabetes mellitus. J Bone Joint Surg Am.

[4] Plotkin LI, Essex AL, Davis HM (2019). RAGE signaling in skeletal biology. Curr Osteoporos Rep.

[5] Cruz NG, Sousa LP, Sousaa MO, Pietrani NT, Fernandesa AP, Gomes KB (2013). The linkage between inflammation and Type 2 diabetes mellitus. Diabetes Res Clin Pract.

[6] Bartell SM, Kim H, Ambrogini E (2014). FoxO proteins restrain osteoclastogenesis and bone resorption by attenuating H₂O₂ accumulation. Nat Commun.

[7] American Diabetes Association. 14 (2018). Diabetes Care in the Hospital: Standards of Medical Care in Diabetes—2018. Diabetes Care.

[8] Barker P, Creasey PE, Dhatariya K (2015). Peri‐operative management of the surgical patient with diabetes. Anaesthesia.

[9] (2020). National Institute for Health and Care Excellence (NICE). NG180: Peri-operative care in adults. Evidence K—Blood glucose control and management. London, UK: NICE. https://www.nice.org.uk/guidance/ng180/evidence/k-blood-glucose-control-management-pdf-317993437910.

[10] (2021). Centre for Perioperative Care; Diabetes UK. Guideline for perioperative care for people with diabetes mellitus undergoing elective and emergency surgery. London, UK: CPOC. https://cpoc.org.uk/sites/cpoc/files/documents/2021-03/CPOC-Diabetes-Guideline2021_0.pdf.

[11] (2018). The National Confidential Enquiry into Patient Outcome and Death (NCEPOD). Highs and lows: A review of the quality of care provided to patients over the age of 16 who had diabetes and underwent a surgical procedure. London, UK: NCEPOD. https://www.ncepod.org.uk/2018pd/Highs%20and%20Lows_Full%20Report.pdf.

[12] Ding ZC, Zeng WN, Rong X, Liang ZM, Zhou ZK (2020). Do patients with diabetes have an increased risk of impaired fracture healing? A systematic review and meta-analysis. ANZ J Surg.

[13] Picke AK, Campbell G, Napoli N, Hofbauer LC, Rauner M (2019). Update on the impact of type 2 diabetes mellitus on bone metabolism and material properties. Endocrinology Connections.

[14] Reich MS, Fernandez I, Mishra A, Kafchinski L, Adler A, Nguyen MP (2019). Diabetic Control Predicts Surgical Site Infection Risk in Orthopaedic Trauma Patients. J Orthop Trauma.

[15] Ahmad MA, Ab Rahman S, Islam MA (2022). Prevalence and Risk of Infection in Patients with Diabetes following Primary Total Knee Arthroplasty: A Global Systematic Review and Meta-Analysis of 120,754 Knees. J Clin Med.

[16] Iorio R, Williams KM, Marcantonio AJ, Specht LM, Tilzey JF, Healy WL (2012). Diabetes mellitus, hemoglobin A1C, and the incidence of total joint arthroplasty infection. The. Journal of arthroplasty.

[17] Kong L, Cao J, Zhang Y, Ding W, Shen Y (2017). Risk factors for periprosthetic joint infection following primary total hip or knee arthroplasty: a meta-analysis. Int Wound J.

[18] Hong SH, Kwon SC, Lee JH, Moon S, Kim JI (2024). Influence of Diabetes Mellitus on Postoperative Complications After Total Knee Arthroplasty: A Systematic Review and Meta-Analysis. Medicina (Kaunas.

[19] Singh JA, Lewallen DG (2013). Diabetes: a risk factor for poor functional outcome after total knee arthroplasty. PLoS One.

[20] Harris AH, Bowe TR, Gupta S, Ellerbe LS, Giori NJ (2013). Hemoglobin A1C as a Marker for Surgical Risk in Diabetic Patients Undergoing Total. Joint Arthroplasty. J Arthroplasty.

[21] Godshaw BM, Ojard CA, Adams TM, Chimento GF, Mohammed A, Waddell BS (2018). Preoperative Glycemic Control Predicts Perioperative Serum Glucose Levels in Patients Undergoing Total. Joint Arthroplasty. J Arthroplasty.

[22] Chrastil J, Anderson MB, Stevens V, Anand R, Peters CL, Pelt CE (2015). Is Hemoglobin A1c or perioperative hyperglycemia predictive of periprosthetic joint infection or death following primary total joint arthroplasty? J Arthroplasty.

[23] Kremers HM, Lewallen LW, Mabry TM, Berry DJ, Berbari EF, Osmon DR (2015). Diabetes mellitus, hyperglycemia, hemoglobin A1C and the risk of prosthetic joint infections in total hip and knee arthroplasty. J Arthroplasty.

[24] Kremers HM, Schleck CD, Lewallen EA, Larson DR, Van Wijnen AJ, Lewallen DG (2017). Diabetes mellitus and hyperglycemia and the risk of aseptic loosening in total joint arthroplasty. J Arthroplasty.

[25] Pomposelli JJ, Baxter JK, Babineau TJ, Pomfret EA, Driscoll DF, Forse RA, Bistrian BR (1998). Early Postoperative Glucose. Control Predicts Nosocomial Infection Rate in Diabetic Patients. Journal of Parenteral and Enteral Nutrition.

[26] Kheir MM, Tan TL, Kheir M, Maltenfort MG, Chen AF (2018). Postoperative Blood Glucose Levels Predict Infection After Total. Joint Arthroplasty. J Bone Joint Surg Am.

[27] Johnston LE, Kirby JL, Downs EA (2017). Postoperative Hypoglycemia Is Associated With Worse Outcomes After Cardiac Operations. Ann Thorac Surg.

[28] Giori NJ, Ellerbe LS, Bowe T, Gupta S, Harris AHS (2014). Many diabetic total joint arthroplasty candidates are unable to achieve a preoperative hemoglobin A1c goal of 7% or less. J Bone Joint Surg Am.

[29] Richards JE, Kauffmann RM, Zuckerman SL, Obremskey WT, May AK (2012). Relationship of hyperglycemia and surgical-site infection in orthopaedic surgery. The. Journal of bone and joint surgery. American volume.

